# Real-Time Monitoring of a Nucleic Acid Amplification Reaction Using a Mass Sensor Based on a Quartz-Crystal Microbalance

**DOI:** 10.3390/bios14040155

**Published:** 2024-03-25

**Authors:** Hideto Kumagai, Hiroyuki Furusawa

**Affiliations:** 1 Graduate School of Organic Materials Science, Yamagata University, Yonezawa 992-8510, Japan; 2 Institute for the Promotion of General Graduate Education (IPGE), Yamagata University, Yonezawa 992-8510, Japan

**Keywords:** polymerase chain reaction, quartz-crystal microbalance, mass sensor, isothermal nucleic acid amplification reaction, recombinase polymerase amplification, DNA fragment detection

## Abstract

Nucleic acid amplification reactions such as polymerase chain reaction (PCR), which uses a DNA polymerase to amplify individual double-stranded DNA fragments, are a useful technique for visualizing the presence of specific genomes. Although the fluorescent labeling method is mainly used with DNA amplification, other detection methods should be considered for further improvements, such as miniaturization and cost reduction, of reaction-monitoring devices. In this study, the quartz-crystal microbalance (QCM) method, which can measure nanogram-order masses, was applied for the real-time detection of DNA fragments in a solution with nucleic acids. This was combined with an isothermal nucleic acid amplification reaction based on the recombinase polymerase amplification (RPA) method, which allowed DNA amplification at a constant temperature. When the DNA amplification reaction was initiated on a QCM sensor plate with an immobilized primer DNA strand, a significant increase in mass was observed compared to when the primer DNA was not immobilized. QCM was shown to be sufficiently sensitive for the in situ detection of amplified DNA fragments. Combining a portable QCM device and RPA offers a sensitive point-of-care method for detecting nucleic acids.

## 1. Introduction

Polymerase chain reaction (PCR), a nucleic acid amplification reaction technique, allows the replication and amplification of individual double-stranded DNA (dsDNA) fragments [1]. PCR has been widely used not only in the field of genetic engineering for the replication of genome fragments [1] but also in the detection of single nucleotide polymorphisms (SNPs) [2], identification of biological species [3], detection of genetically modified organisms [4], and detection of bacteria and viruses related to infectious diseases [5,6]. Recently, PCR has been used to detect environmental DNA to monitor organisms in water and other environments [7,8]. Circulating tumor DNA (ctDNA) in a patient’s blood and urine, which can be visualized by PCR amplification, could be a biomarker for the recurrence of cancer [9,10]. Therefore, PCR is the gold standard for nucleic acid amplification reaction techniques and essential in bioscience and biosensing fields.

The PCR procedure is as follows: (1) a region of replication in the source dsDNA, called the template, is determined; (2) a short single-stranded DNA (ssDNA), called primer DNA, which is approximately 20–30 nt long, is prepared, with each primer having complementary sequences to both 3′ ends of each DNA strand of the replication region; (3) DNA polymerase induces DNA elongation from each primer DNA bound to the template DNA in a sequence-dependent manner; (4) the primer DNA then binds the replication product DNA, and another strand is elongated; and (5) by repeating steps (3) and (4), the DNA fragments in the region sandwiched by the primer DNA are amplified exponentially [1]. 

Generally, PCR devices require a module that can quickly change the temperature. This is because, in step (3), the system must be heated to above 90 °C to denature the template dsDNA into two ssDNAs and then cooled to a lower temperature (approximately 50 °C) to bind the primer DNA to the template. In addition, because fluorescently labeled DNA is used to detect the amplified DNA fragments in step (5), the PCR device is equipped with an optical module to measure the change in fluorescence. Considering the need to incorporate a temperature controller, called a thermal cycler, and optical devices, PCR instruments are predominantly used as benchtop devices in research institutes. In addition, small microfluidic-based PCR devices for point-of-care (POC) applications have been developed and studied [11]. To further improve the development of POC gene detection devices, combinations of nucleic acid amplification techniques that differ from conventional PCR with other detection methods should be considered.

Isothermal nucleic acid amplification reaction methods, such as loop-mediated isothermal amplification (LAMP), helicase-dependent amplification (HDA), strand displacement amplification (SDA), rolling circle amplification (RCA), and recombinase polymerase amplification (RPA), do not require a thermal cycler [12,13]. Methods for detecting DNA products include optical sensors, such as surface plasmon resonance (SPR) sensors, or electrochemistry-based biosensors, such as amperometry or differential pulse voltammetry [12]. However, LAMP, SDA, and HDA methods require high temperatures of 60–65 °C for the reaction. Although RCA can be performed at ~30 °C, it is not suitable as a replacement for a conventional PCR method because the product is DNA strands that are longer than the template DNA, which differs from a conventional PCR product. 

We focused on the RPA method because the reaction can be performed at approximately 37 °C, which is slightly higher than room temperature, and the reaction process is similar to that of conventional PCR [14]. The RPA method is characterized by its use of recombinase and single-stranded DNA-binding protein (SSB). In conventional PCR, the binding of primer DNAs to a template DNA is made possible by a temperature change from above 90 °C to approximately 50 °C in step (3). In the RPA method, the recombinase first binds to the primer DNA, and then the complex binds to the complementary sequence region of the template dsDNA to form a ssDNA-recombinase–dsDNA complex [14,15,16]. After complementary strand recombination, SSB binds to the free ssDNA, and an elongation reaction occurs from the primer DNA on the template by DNA polymerase. Because these processes occur at ~37 °C, they proceed isothermally and the amplified product is also a DNA fragment from the region sandwiched by the primers. Therefore, RPA is referred to as isothermal PCR. The requirement for relatively low temperatures is advantageous for use in combination with various sensor devices other than optical or electrochemical biosensors. The similarity of the reaction process to conventional PCR is also advantageous in that it can be used in the same manner as PCR for all previous applications.

A quartz-crystal microbalance (QCM) is a mass sensor using a quartz-crystal oscillator [17,18,19,20]. When a substance is adsorbed onto a sensor surface, the frequency (*F*) decreases according to the mass of the adsorbed substance. In the case of the 27 MHz oscillator, the frequency decreases by 1 Hz for a mass increase of 0.6 ng/cm^2^. Thus, the QCM sensor can be used to observe mass changes at the nanogram level [21,22]. Previously, we applied a QCM as a biosensor, in which a receptor was immobilized on the sensor surface and oscillated in water and the receptor’s capture/release of the target molecule was observed by the mass increase/decrease calculated from the frequency changes (∆*F*) [23]. Using the QCM on which a template DNA and primer DNA complex were immobilized, the binding of Klenow fragments with the activity of DNA polymerase on the protruding end of the DNA and then the elongation reaction upon the addition of dNTPs could be observed as mass changes on the sensor surface [21]. 

**Figure 1:**
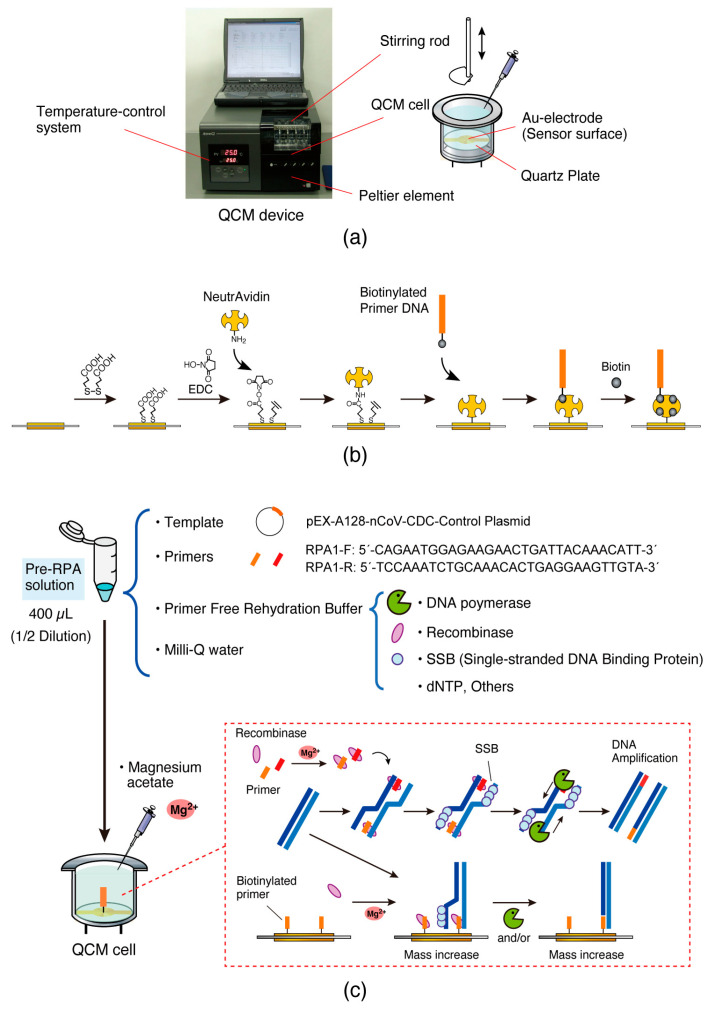
Schematic illustrations of (**a**) a 27 MHz QCM device equipped with a temperature-control system and a QCM sensor cell, (**b**) the process of primer DNA immobilization on a QCM sensor surface, and (**c**) RPA reaction monitoring in a QCM cell.

Despite the sensitivity of the QCM biosensor in detecting DNA polymerase reactions, the very large background noise caused by temperature changes makes it difficult to adapt QCM to conventional PCR monitoring. In contrast, it is expected that RPA (isothermal PCR) without temperature changes can be monitored using QCM. In this study, we monitored the amplification of DNA fragments in RPA as a mass change on a QCM sensor plate (Figure 1). One of the two primer DNAs was immobilized on the QCM plate, which was filled with pre-RPA reaction solution, and then magnesium ions, which are essential for the reaction, were added while monitoring the frequency changes (∆*F*). We previously reported that QCM devices can be miniaturized to the integrated circuit (IC) card size [24]. Successful monitoring of DNA amplification reactions on a QCM could enable the development of gene detection devices that employ inexpensive and simple oscillator circuits. These devices could be used in homes and small clinics for POC applications. This study aimed to advance the fabrication of portable gene detection devices.

## 2. Materials and Methods

### 2.1. Materials

Primer DNAs (30 mer ssDNA) needed for PCR and biotinylated primer DNA (30 mer) were custom-synthesized by Eurofins Genomics, K.K. (Tokyo, Japan). pEX-A128-nCoV-CDC-Control Plasmid DNA, which contains an internal gene as a model PCR template (338 bp), was purchased from Eurofins Genomics, K.K. (Tokyo, Japan). The chemical reagents 3,3-dithiodipropionic acid (Nacalai Tesque, Kyoto, Japan), 1-ethyl-3-[3-(dimethylamino)propyl]carbodiimide (EDC) (Dojindo Laboratories, Kumamoto, Japan), *N*-hydroxysuccinimide (NHS) (Wako Pure Chemical Industries, Ltd., Osaka, Japan), and NeutrAvidin (Thermo Fisher Scientific Inc., Tokyo, Japan) were used for experiments of DNA modification to a sensor surface. NaCl (Wako Pure Chemical Industries, Ltd., Osaka, Japan), tris(hydroxymethyl)aminomethane (Wako Pure Chemical Industries, Ltd., Osaka, Japan), 2-[4-(2-hydroxyethyl)-1-piperazinyl]ethanesulfonic acid (HEPES) (Dojindo Laboratories, Kumamoto, Japan), ethylenediamine-*N,N,N’,N’*-tetraacetic acid, disodium salt (EDTA·2Na) (Dojindo Laboratories, Kumamoto, Japan), and TBE (Tris-borate-EDTA) powder (TaKaRa Bio Inc., Shiga, Japan) were used to prepare buffer solutions. A TwistAmp Basic Kit (TwistDx Ltd., Maidenhead, UK) was used for isothermal PCR based on the RPA method. Agarose L03 (TaKaRa Bio Inc., Shiga, Japan), Novel Juice (The BIO-HELIX Co., LTD., New Taipei City, Taiwan) as a DNA staining solution, and a 100 bp DNA Ladder (TaKaRa Bio Inc., Shiga, Japan) were used for DNA electrophoresis. Ultrapure water (Milli-Q) was used for all the experiments.

### 2.2. QCM Device

An AFFINX Q4 QCM instrument (Initium, Inc., Tokyo, Japan) was used in this study [23]. Because the QCM device was equipped with stirring rods and a temperature-control system (setting range: 10–40 °C; resolution: ±0.1 °C), the solution in the QCM sensor cell could be maintained at a constant condition (Figure 1a). At the bottom of the sensor cell, there was a 27 MHz QCM plate (8.7 mm diameter) with an Au-electrode (5.7 mm^2^). The sensor cell was 500 µL in volume and covered with a 0.5 mm thick urethane gel sheet to avoid the loss of the reaction solution through evaporation (Exseal Co., Ltd., Gifu, Japan). The sheet, which was made in our laboratory for the experiment, had a sample inlet and a hole that did not interfere with the stirring rod. The quartz crystals in the sensor cell were oscillated at 27 MHz using an oscillation circuit set in the device.

### 2.3. RPA Reaction Design

To demonstrate the RPA reaction in a QCM cell, DNA primers were designed for the amplification of a DNA fragment (201 bp) from an internal gene (338 bp) of a pEX-A128-nCoV-CDC-Control Plasmid. The designed DNA sequences of the forward and reverse primers were as follows: RPA1-F (5′-CAGAATGGAGAAGAACTGATTACAAACATT-3′, 30 mer) and RPA1-R (5′-TCCAAATCTGCAAACACTGAGGAAGTTGTA-3′, 30 mer), respectively. Biotinylated RPA1-F was prepared as the primer DNA to be displayed on the QCM sensor surface.

### 2.4. Immobilization of DNA Primer to QCM Sensor

Biotinylated primer DNA was immobilized on the Au electrode of the QCM cell as described previously [24]. Briefly, the Au electrode was modified with 3,3-dithiodipropionic acid through Au-S interactions (Figure 1b). A mixture of EDC and NHS was drop-cast onto the modified Au electrode to activate carboxylic acids. Subsequently, the sensor cell was filled with 500 µL of HEPES buffer solution (10 mM HEPES-NaOH, pH 7.9, 0.2 M NaCl) and then set in the QCM device. After the reaction of NeutrAvidin with the activated ester, the solution in the QCM cell was replaced by Tris buffer solution (10 mM Tris-HCl, pH 8.0, 1 mM EDTA, 0.15 M NaCl), and the biotinylated primer DNA was then injected into the cell with real-time monitoring of the QCM frequency change (∆*F*). The amount of immobilization of the primer DNA via the avidin–biotin interaction was controlled by the addition of free biotin when the predetermined amount of immobilization was reached with a −100 Hz frequency change.

### 2.5. Preparation of the RPA Reaction Solution

An RPA reaction solution was prepared using a commercially available TwistAmp Basic Kit, in which a “Primer Free Rehydration” buffer containing the factors required for the RPA reaction, such as recombinase, SSB, and dNTP, was included (Figure 1c). The reaction solution, which was diluted to half with Milli-Q water against the concentration indicated in the manufacturer’s instructions, was used to monitor the performance of the RPA reaction in the QCM cell, leveraging the stable oscillation of the quartz plate during monitoring within the reaction solution, which experienced decreasing viscosity. In total, 400 µL of the solution contained the following: 9.6 µL each of a 10 µM DNA solution for RPA1-F and RPA1-R primers, 118 µL of “Primer Free Rehydration” buffer, 4.0 µL of 10^5^ units/µL (170 fM) of the template plasmid solution, and 248.8 µL of Milli-Q water. The pre-RPA solution used in this study contained no magnesium ions (Mg^2+^) essential for RPA reactions. The magnesium acetate solution supplied in the kit was used to initiate the reaction according to the manufacturer’s instructions.

### 2.6. Observation of RPA Reaction on QCM

The pre-RPA solution was added to the QCM cell, in which the primer DNA was immobilized on the Au electrode. The QCM cell was placed in the QCM device and covered with a custom-made urethane gel sheet. After setting the temperature around the QCM cell to 35 °C and confirming that the oscillation frequency of the quartz plate was stabilized, 10 µL of a 280 mM magnesium acetate solution was injected into the cell to start the RPA reaction. The RPA reaction on the QCM sensor plate was measured as frequency change (∆*F*) over time. Unless otherwise stated, the experimental conditions were as follows: [Mg^2+^] = 7 mM, [Primer] = 0.24 µM, [Template] = 1.7 fM, RPA solution: 1/2 dilution at 35 °C.

### 2.7. Confirmation of RPA Products by Electrophoresis

After an RPA reaction on the QCM plate, 100 µL of the solution in the QCM cell was collected. The solution was then purified using a NucleoSpin Gel and a PCR Clean-up kit (TaKaRa Bio Inc., Shiga, Japan), which allowed the purification of DNA products from RPA solutions by removing proteins using a spin column method (Figure S1). The purified DNA solution was mixed with Novel Juice as a staining reagent and analyzed by electrophoresis on a 3% agarose gel in TBE Buffer with Mupid-2plus (TaKaRa Bio Inc., Shiga, Japan). After electrophoresis, the gel was photographed using a Gel Doc EZ Imaging System (Bio-Rad Laboratories Inc., Hercules, CA, USA).

### 2.8. Quantification of RPA Products by Fluorescence Measurements

The QCM cell was filled with 400 µL of the pre-RPA solution, covered with a custom-made urethane gel sheet, and set in the device at a controlled temperature of 35 °C. The stirring function of the device was not used. After the RPA reaction began with an injection of 10 µL of a 280 mM magnesium acetate solution, 10 µL of the reaction solution was sampled from the QCM cell every 2 min. Each 8 µL of sampled solution was immediately mixed with 16 µL of a quench solution (10 µL of 500 mM EDTA-Na, pH 7.9, 4 µL of Novel Juice, and 2 µL of Milli-Q water) to stop the reaction. To visualize the DNA products, each 6 µL of the quenched solution was applied to wells of a 3% agarose gel and imaged using a Gel Doc EZ Imaging System without electrophoresis. The amount of dsDNA was determined by comparison with a dilution series of the DNA ladder with a known amount of dsDNA. The fluorescence intensity in the obtained images was quantified by the accompanying software.

## 3. Results and Discussion

### 3.1. RPA Reaction Monitoring on a QCM Plate

**Figure 2:**
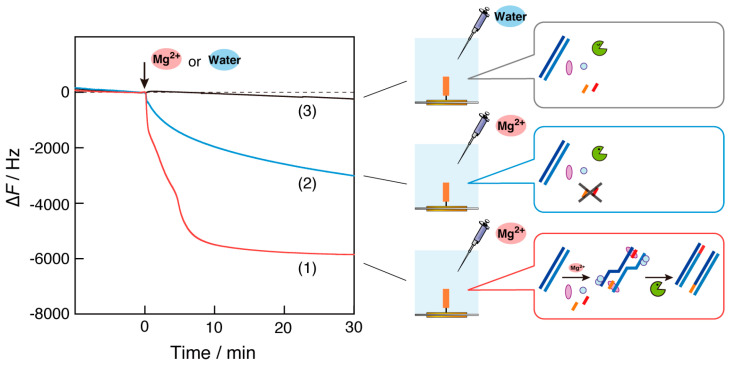
Time courses of the frequency changes (∆*F*) of RPA reaction monitoring in response to the addition of magnesium acetate (Mg^2+^ ions) into the QCM cell filled with (1) RPA reaction solution and (2) the solution in the absence of DNA primers, and (3) in response to the addition of water instead of the Mg^2+^ ion solution. Repetition (*n* = 3) of curve (1) resulted in a mean of −5800 Hz and standard deviation of 210 Hz for the frequency response at 20 min.

The mass change on the sensor surface of the QCM plate during the RPA reaction in the QCM cell filled with the pre-RPA solution was measured in response to the addition of Mg^2+^ (Figure 2, curve 1). The frequency of the QCM decreased immediately after the addition of Mg^2+^ ions to approximately ∆*F* = −2000 Hz, continued to slightly decrease to approximately −4000 Hz, and then decreased again quickly to approximately −6000 Hz and remained constant. The three steps observed could be considered as follows: (step 1) the recombinase binds to the primer DNA on the QCM plate due to activation by Mg^2+^ ions; (step 2) the viscosity of the solution increases as the RPA reaction progresses because of the exponentially amplified DNA fragments in the bulk; and (step 3) the amplified DNA fragments form a complex of ssDNA-recombinase-dsDNA through a primer DNA on the QCM plate and/or an elongation reaction is triggered by DNA polymerase (see Figure 1c). Steps 2 and 3 could occur in any order.

This hypothesis is supported by the result of Figure 2, curve 2, in which Mg^2+^ ions were added to the pre-RPA solution in the absence of DNA primers. The observed reduction in frequency (mass increase) was a simple one-step curve. This result is consistent with the expectation that an RPA reaction would not occur in solution and that the recombinase would bind to the primer DNA on the QCM plate. Thus, the simple one-step decreasing curve for ∆*F* should correspond only to step 1 of curve 1. In addition, frequency reduction can occur when the viscosity of the solution increases, owing to the mechanical load mass in the quartz-crystal oscillation [22]. The fact that the increase in DNA fragments caused an increase in viscosity and resulting decrease in frequency is consistent with the fact that curves 2 and 3, in which DNA fragments were not amplified, showed no decrease in frequency, corresponding to step 2 of curve 1.

As shown in curve 3, the frequency at which no Mg^2+^ ions were injected into the QCM cell exhibited a negligible change, with only a little drift. This result indicates that the QCM measurements could be carried out on a stable baseline, even under conditions of 35 °C, which is different from the normal measurements at 20–25 °C and under conditions of high viscosity of the RPA solution.

### 3.2. Effect of Primer DNA Displayed on the Plate

**Figure 3:**
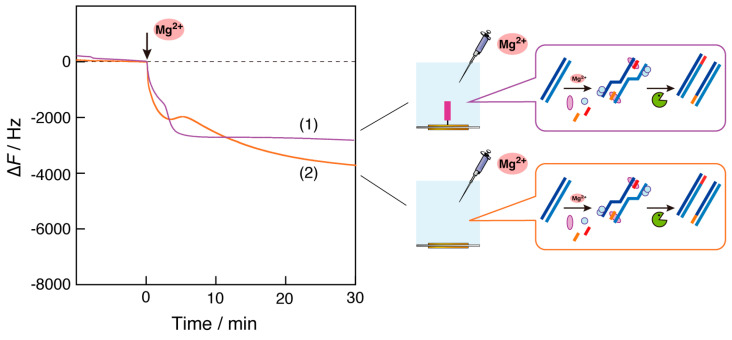
Time courses of the frequency changes (∆*F*) of RPA reaction monitoring on the QCM plate (1) when non-primer DNA was immobilized and (2) when no primer DNA was immobilized, responding to the addition of Mg^2+^ ions into the QCM cell. The sequence of non-primer DNA: bio-5′-CGCCCCACGTAAAGCGACTAAAACCCCAGG-3′.

To examine the effect of primer DNA immobilized on the QCM plate, we observed RPA reactions on the plate where non-primer DNA was immobilized (Figure 3, curve 1). The sequence of the non-primer DNA was designed to be different from that of the bulk primer DNAs (RPA1-F and -R). After the addition of the Mg^2+^ ion solution, a two-step reduction in frequency was observed. The first decrease in frequency can be explained by the binding of recombinase to non-primer DNA because the first binding of recombinase to ssDNA is sequence-independent. The explanation that the second reduction is due to the increased viscosity as the RPA reaction progresses is consistent with the results shown in Figure 2, although the change was small.

Curve 2 in Figure 3 shows the results of the RPA reaction on the QCM plate, where no primer DNA was immobilized. We expected to observe no decrease in frequency due to the absence of recombinase binding in the first step and only a reduction in the frequency of the increased viscosity caused by increased dsDNA fragments with the RPA reaction in progress. However, the observed curve included three steps: a frequency decrease (mass increase) between 0 and 2 min; a frequency increase (mass decrease) between 2 and 5 min; and a frequency decrease after 5 min (curve 2). A possible explanation is that the complex of DNA primers in the bulk and recombinase activated by Mg^2+^ ions was adsorbed on the QCM plate without DNA displayed in the first 0–2 min, the complex was desorbed on the plate due to binding to the amplified DNA fragments in the bulk, and finally, dsDNA amplification that caused enough viscosity to decrease the frequency of the QCM oscillation occurred. 

Considering the results in Figure 3, we found that when the same DNA primer used for the RPA reaction was immobilized on the QCM plate, a large frequency decrease (mass increase) was observed, as shown by curve 1 in Figure 2.

### 3.3. Factor-Separation Experiments

**Figure 4:**
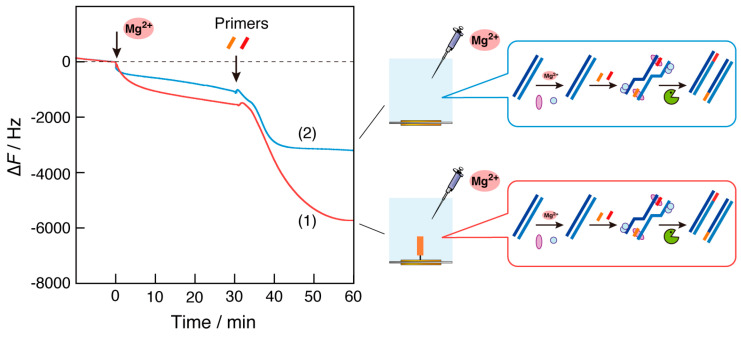
Time courses of the frequency changes (∆*F*) of RPA reaction monitoring on the QCM plate (1) when the primer DNA was immobilized and (2) when no primer DNA was immobilized in response to the addition of Mg^2+^ ions and the DNA primers were injected separately into the QCM cells.

In biosensors, a batch-type measurement cell, rather than a flow-type cell, allows for the separate addition of reaction factors. It has been useful for investigating the mechanism of biomolecular complex formation [25]. We performed factor-separation experiments in which Mg^2+^ ions and DNA primers were injected sequentially into the cells (Figure 4). When Mg^2+^ ions were injected onto the QCM plate, the primer DNA was immobilized, and a mass increase (frequency decrease of approximately 2000 Hz) due to the binding of recombinase activated by Mg^2+^ ions to the immobilized primer DNA was observed (curve 1). Following the addition of the primer DNAs, a large decrease in frequency (approximately −4000 Hz) was observed after a short time lag. It is reasonable to assume that this large frequency change corresponds to both the increase in bulk viscosity associated with the RPA reaction and the binding of the amplified DNA fragments to the primer on the QCM plate, as shown by curve 1 in Figure 2. 

In the case of no primer DNA on the QCM plate (curve 2), a slight increase in the mass was observed after the addition of Mg^2+^ ions, followed by a moderate change in frequency with the addition of primer DNAs (approximately −2000 Hz). From a comparison with the initial part (0–2 min) of curve 2 in Figure 3, we determined that the primer DNA in the bulk and the Mg^2+^-activated recombinase complex would be adsorbed on the QCM plate non-specifically.

The difference in the frequency decrease between curves 1 and 2 after the injection of the primer DNA could be attributed to the formation or non-formation of a complex between the primer DNA on the plate and the amplified dsDNA in the bulk. Thus, the use of a mass sensor with immobilized primer DNA makes it possible to capture the amplified DNA product and monitor the RPA reaction as the mass changes.

### 3.4. Concentration and Temperature of RPA Reaction Solution for QCM Measurements

**Figure 5:**
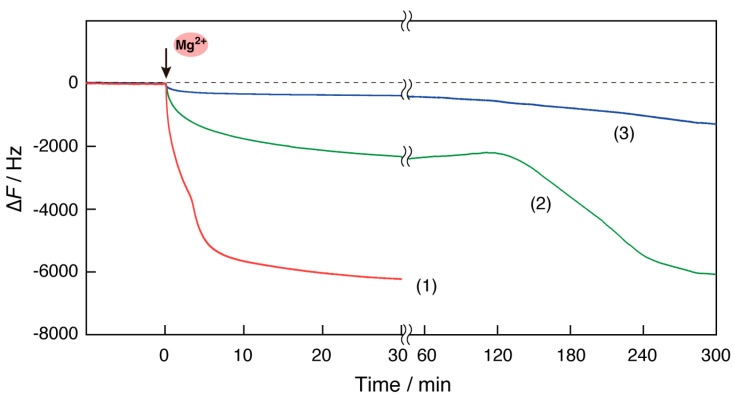
Time courses of the frequency changes (∆*F*) of RPA reaction monitoring in RPA reaction solution diluted to (1) 1/2, (2) 1/4, and (3) 1/8 using Milli-Q-water. The experimental conditions: (1) [Mg^2+^] = 7 mM, [Primer] = 0.24 µM, (2) [Mg^2+^] = 3.5 mM, [Primer] = 0.12 µM, and (3) [Mg^2+^] = 1.75 mM, [Primer] = 0.06 µM at 35 °C.

Because a crowding reagent such as a hydrophilic polymer was included in the reaction solution of the RPA kit used in this study [14], measurements with QCM oscillation were not suitable with the highly viscous solutions. In fact, the measurement conditions indicated in the TwistDx kit manual could not be used owing to significant signal drift. To avoid high viscosity, an RPA solution diluted with Milli-Q water was used. As shown in Figure 5 (curve 1), the RPA reaction in the 1/2 dilution of the original solution indicated a quick response within 5 min and a significant signal change on the QCM plate. In contrast, the frequency decrease in the case of the 1/4 diluted solution was similar to that of the 1/2 diluted solution; however, the reaction time was approximately 24 times longer than that of the 1/2 diluted solution (curve 2). Furthermore, the use of the 1/8 dilution resulted in small signal changes. These results indicate that observation of the RPA reaction is possible up to 1/4 dilution and that an RPA concentration of 1/2 dilution is reasonable for DNA detection on the QCM plate.

As for the reaction temperature of RPA, a range of 22–45 °C is possible, with temperatures of 37–42 °C considered optimal [14]. In contrast, the QCM is designed to provide stable measurements at 20–25 °C. To determine the optimal reaction temperature, the RPA reaction products at 25–40 °C in microtubes were confirmed by electrophoresis (Figure S1a). The reaction products at 25 °C and 30 °C indicated DNA of less than the expected length (<201 bp). At 35 °C and 40 °C, the expected length of DNA was amplified. Considering the stability of the QCM measurement, we determined that 35 °C was the optimal temperature for the RPA reaction on the QCM plate. To confirm the RPA status in the QCM cell at 35 °C, after monitoring the RPA reaction, the reaction solution in the cell was collected to confirm the amplified DNA fragments by electrophoresis (Figure S1b). Electrophoresis of the QCM cell solution, in which the RPA reaction was triggered by the addition of Mg^2+^ ions, revealed DNA fragments of the expected length (201 bp). 

### 3.5. Identification of the Cause of the Mass Increase on the QCM Plate

**Figure 6:**
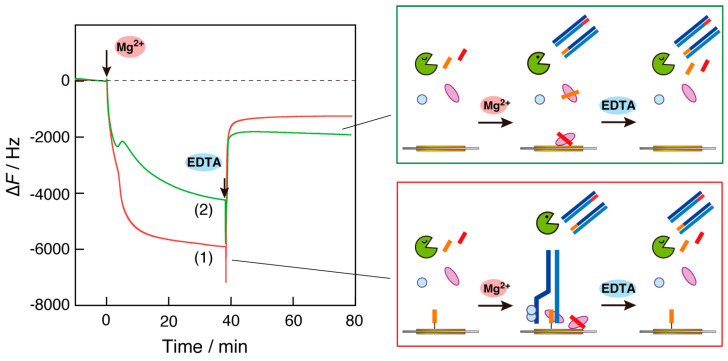
Time courses of the frequency changes (∆*F*) of RPA reaction monitoring with the addition of Mg^2+^ ions and after resetting monitoring with the addition of EDTA solution on the QCM plate (1) when primer DNA was immobilized and (2) when no primer DNA was immobilized. The experimental condition: [EDTA] = 100 mM.

The progression of the RPA reaction could be detected as a large frequency decrease (approximately ∆*F* = −6000 Hz) on the QCM plate (see Figure 2, curve 1). Based on a comparison with the curves in Figure 3, monitoring step 3 is particularly important, which is considered to correspond to (1) the formation of a complex of ssDNA-recombinase-dsDNA with amplified DNA fragments and primer DNA on the plate and/or (2) the elongation reaction caused by DNA polymerase from the primer DNA on the plate (Figure 1c). We expected the elongation reaction on the plate to occur as a result of DNA polymerization, advancing alongside the RPA reaction. To clarify the events involved in this step, two confirmatory experiments were conducted. First, EDTA addition experiments were performed (Figure 6). After the RPA reaction in the QCM cell, EDTA was added at a final concentration of 100 mM to capture Mg^2+^ ions, and the conditions were reset to those prior to Mg^2+^ addition. As shown in Figure 6, the frequency increased from −6000 Hz to approximately −1500 Hz after the EDTA addition (curve 1). Even in the absence of primer DNA on the plate, the frequency increased from −4000 Hz to a comparable level (curve 2). The similar frequency levels on the QCM with and without the primer DNA indicated that the DNA elongation from the primer on the plate should not occur and that the amplified DNA fragments could form a paperclip-like complex, which would be decomposed by EDTA and dissociated from the plate. This suggestion was supported by a second confirmatory experiment, DNA hybridization (Figure S2). The QCM cells, in which the RPA reaction solution containing EDTA (Figure 6) was replaced with a fresh solution, followed by the sequential addition of three complementary DNAs (A: 15 mer cDNA for the lower side of the primer DNA, B: 15 mer cDNA for the upper side of the primer DNA, and C: 15 mer cDNA for the expected elongation DNA). Because the primer DNA (30 mer) on the plate was immobilized at −100 Hz, the 50 Hz frequency decreases with the addition of cDNAs (A) and (B) were reasonable (Figure S2, curve 1). In contrast, the addition of cDNAs (C) did not change the frequency. This result indicated that DNA elongation did not occur on the quartz plate. Thus, we concluded that the successful observation of the RPA reaction by mass changes was due to the formation of a recombinase complex via primer DNA anchored on the QCM plate, similar to a paperclip (Figure S3), as the complex can reversibly bind to and dissociate from the primer DNA.

Compared to detection by an elongation reaction using DNA polymerase, detection of dsDNA by formation of the paperclip-like complex using recombinase may have advantages in terms of sequence specificity, speed of reaction, and potential for repeated use of the sensor.

### 3.6. Estimation of Detected Concentrations of RPA Products

**Figure 7:**
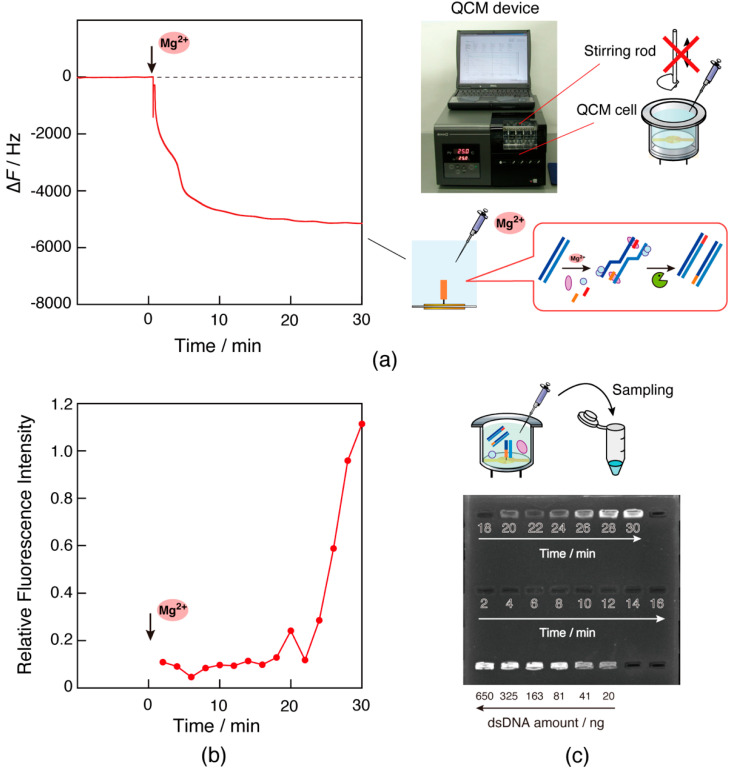
(**a**) Time courses of the frequency changes (∆*F*) of RPA reaction monitoring in response to the addition of Mg^2+^ ions using pipetting rather than stirring in the QCM device. Repetition (*n* = 3) of the curve resulted in a mean of −4900 Hz and standard deviation of 130 Hz for the frequency response at 20 min. (**b**) Plots of fluorescence intensity when the dsDNA staining agent was mixed with the reaction solution and sampled from a QCM cell every 2 min and (**c**) an image from the fluorescence analysis. The relative fluorescence intensity of the samples was calculated using the fluorescence intensity of 81 ng of dsDNA amount as 1.

RPA combined with a fluorophore-quencher detection method can be used to detect reaction products within 15–30 min from 10^5^–10^2^ copies of a template [26]. The QCM detection method, however, showed distinguishable changes within 10 min from 4 × 10^5^ copies of a template, compared with a different template that did not match the primer DNAs (Figure S4). We confirmed the amplification rate by sampling the reaction product in the QCM cell over time (Figure 7). To allow sampling, the stirring function of the device was not used. As a preliminary experiment for comparison under the same conditions, QCM measurements were obtained without using the stirring rod but instead using a pipetting apparatus (Figure 7a). Quantification with a fluorescent stain for dsDNA visualization showed 18 ng/µL (133 nM) of product at 26 min after the start of the reaction (Figure 7b,c). This result means there was an approximately 10^8^-fold increase from 1.7 fM of the template DNA. The estimated doubling time was calculated to be 1.0 min. The detectable concentration of the QCM device should be calculated as 1.8–7.2 pM, based on the doubling volume after 10–12 min. However, there is a previous report of 10^4^-fold amplification within 10 min, that means a doubling time of 0.75 min [27]. The doubling time could be not constant throughout the reaction and may be short in the initial phase. In that case, the detectable concentration was estimated to be 18–117 pM. In either case, the ability to detect DNA at low pM levels may be due to the high stability of the paperclip-like complex. Therefore, combination of the QCM method with an RPA reaction allowed for more rapid dsDNA detection.

### 3.7. RPA Reaction Detection in a Lab-Made QCM Device

**Figure 8:**
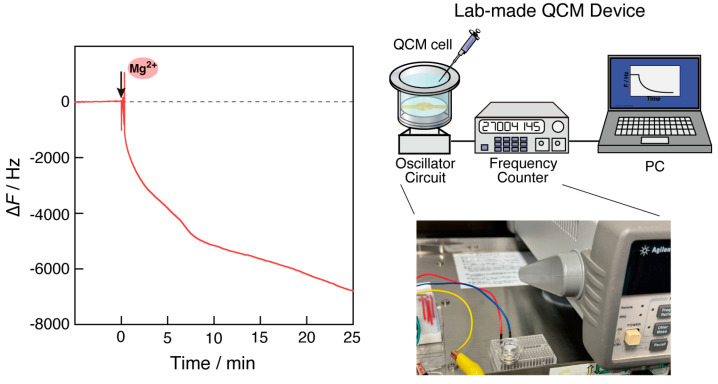
Time courses of the frequency changes (∆*F*) in RPA reaction monitoring in response to the addition of Mg^2+^ ions without using pipetting rather than stirring in the custom-made QCM device.

To inform the development of future portable gene detection devices, we tested whether the RPA reaction could be observed in a small QCM device without stirring. Measurement without using the stirring function, as is the case when using benchtop devices, is indicated in Figure 7a. Although the frequency was disordered after the addition of Mg^2+^ ions and the pipetting operation, a large frequency decrease above −5000 Hz was observed, including three step changes similar to that shown in curve 1 in Figure 2. The reaction time was not significantly different from that in the case with stirring rods, compared to curve 1 in Figure 2. Similar results were obtained in repeated experiments, respectively (Figure S5). This suggests that the reaction can be observed without stirring. Moreover, the experiment without stirring was carried out using a lab-made instrument built with an oscillator circuit assembled on a breadboard, a QCM cell, and a general-purpose frequency counter in a thermostatic chamber at 35 °C (Figure 8). Although disorders in the frequency signal were observed in response to the pipetting operation after the addition of Mg^2+^ ions, a multi-step curve in excess of −5000 Hz was observed. Frequency drift was also observed after 10 min owing to non-optimization for viscosity in the lab-made device. Although further optimization of the drive circuit is required, these results suggest the possibility of gene detection using a QCM sensor.

## 4. Conclusions

We investigated the possibility of tracking PCR reactions in real-time using a mass sensor based on QCM. Although QCM is disadvantageous with stable oscillation at high temperatures and for viscous solutions, isothermal PCR based on the RPA method could be performed at 35 °C and the 1/2-diluted reaction solution allowed the RPA reaction and QCM measurement to proceed together. By combining the QCM and RPA methods, we could detect amplified DNA fragments within 10 min in a DNA sequence-specific manner, with no fluorescent labeling under the conditions of this experiment. The QCM device is expected to be miniaturized because it simply consists of an oscillation circuit and a frequency counter that can be as small as an IC card. Although an oscillator circuit suitable for high-viscosity solutions is required, we believe that the technique used here for the observation of the RPA reaction with QCM will form the basis for a novel method of gene detection.

## Data Availability

The data presented in this study are available on request from the corresponding author.

## Supplementary Materials

The following supporting information can be downloaded at: https://www.mdpi.com/article/10.3390/bios14040155/s1, Figure S1: schematic illustrations and electrophoresis images for the confirmation of RPA reaction products; Figure S2: confirmation of DNA elongation and non-elongation by DNA hybridization experiments; Figure S3: image of a paperclip; Figure S4: QCM measurement for RPA reaction monitoring with a template DNA matching the primers or a template DNA that did not match the primers; Figure S5: repetitive validation of QCM measurements for RPA reaction monitoring.
