# Supplementary material for: Real-Time Monitoring of a Nucleic Acid Amplification Reaction Using a Mass Sensor Based on a Quartz-Crystal Microbalance
Source: Biosensors (Basel). 2024 Mar 25;14(4):155. doi: 10.3390/bios14040155 (PMC11048521; doi:10.3390/bios14040155)
Supplement: Supplementary file 1 [file biosensors-14-00155-s001.zip › biosensors-2871841-supplementary.pdf]

Real-Time Monitoring of a Nucleic Acid Amplification Reaction Using a Mass Sensor Based on a Quartz-Crystal Microbalance

Hideto Kumagai[1] and Hiroyuki Furusawa[1,2]*

[1]Graduate School of Organic Materials Science, Yamagata University, Yonezawa 992-8510, Japan

[2]Institute for the Promotion of General Graduate Education (IPGE), Yamagata University, Yonezawa 992-8510, Japan

*Correspondence: hfurusaw@yz.yamagata-u.ac.jp; Tel.: +81-238-26-3841

Supporting Information

Table of Contents

Figure S1: Schematic illustrations and electrophoresis images for the confirmation of RPA reaction products.

Figure S2: Confirmation of DNA elongation and non-elongation by DNA hybridization experiments.

Figure S3: Image of a paperclip.

Figure S4: QCM measurement for RPA reaction monitoring with a template DNA matching the primers or a template DNA that did not match the primers.

Figure S5: Repetitive validation of QCM measurements for RPA reaction monitoring.

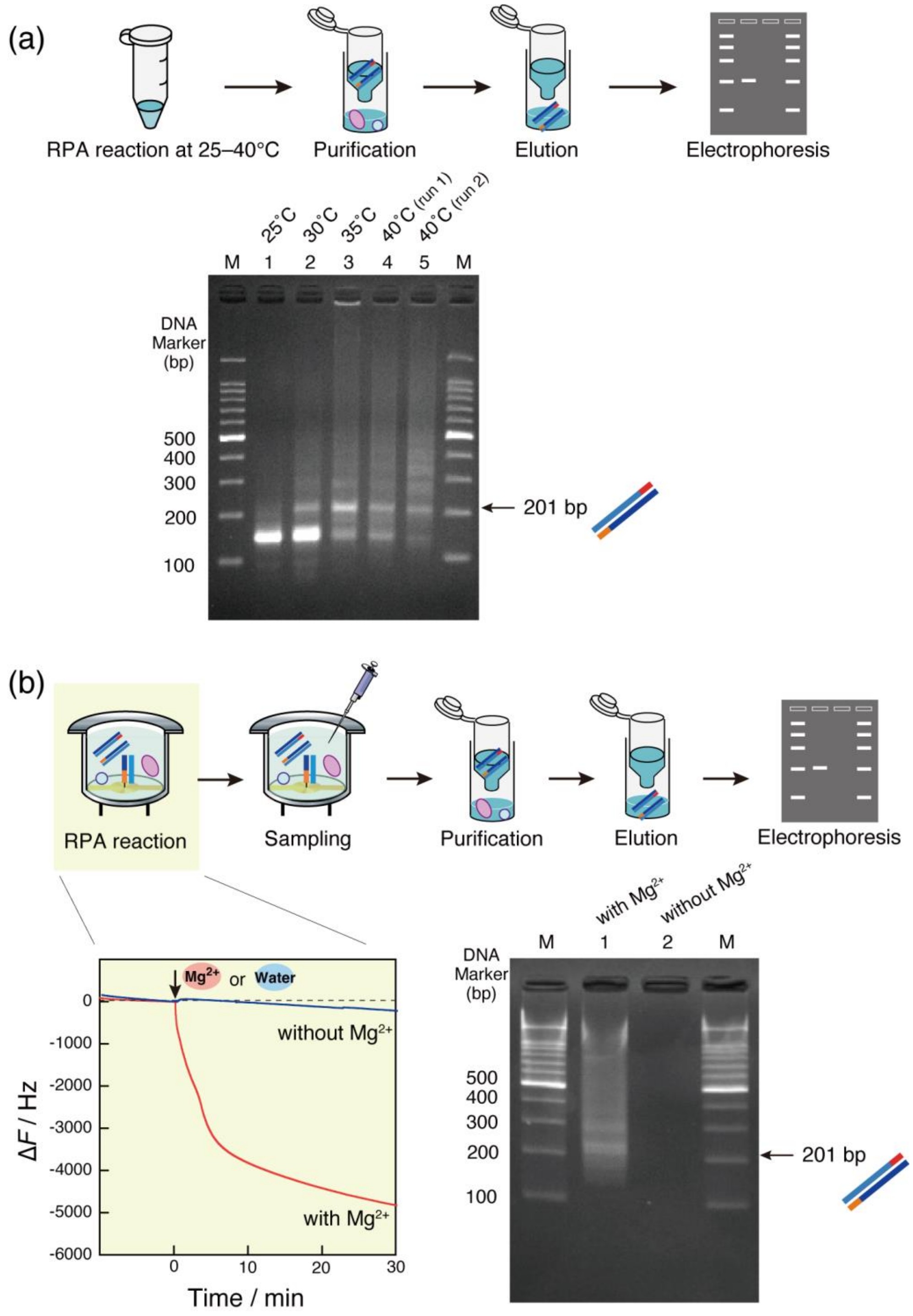

Figure S1. Schematic illustrations and electrophoresis images for the confirmation of (a) effect of RPA reaction temperature and (b) RPA reaction products in the QCM cell.

Experimental Information:

(a) RPA reaction was carried out using TwistAmp Basic Kit. The reaction solution was diluted to half by Milli-Q water against the concentration indicated in the kit manual. Each 100 μL of solution in a microtube was set in a thermal cycler device (PC-707, ASTEC Co., Ltd., Japan) and the temperature was controlled at

25˚C, 30˚C, 35˚C, and 40˚C, respectively. After the reaction of 30 min, the solution in each microtube was purified using a spin column method, and analyzed by electrophoresis (see section 2.7. Confirmation of PCR products by electrophoresis" in the text.

(b) The RPA reaction in the QCM cell was performed as mentioned in the text (see section 2.6 Observation of RPA reaction on QCM). After the reaction, the solution in the cell was collected, followed by purification using a spin column method, and analyzed by electrophoresis.

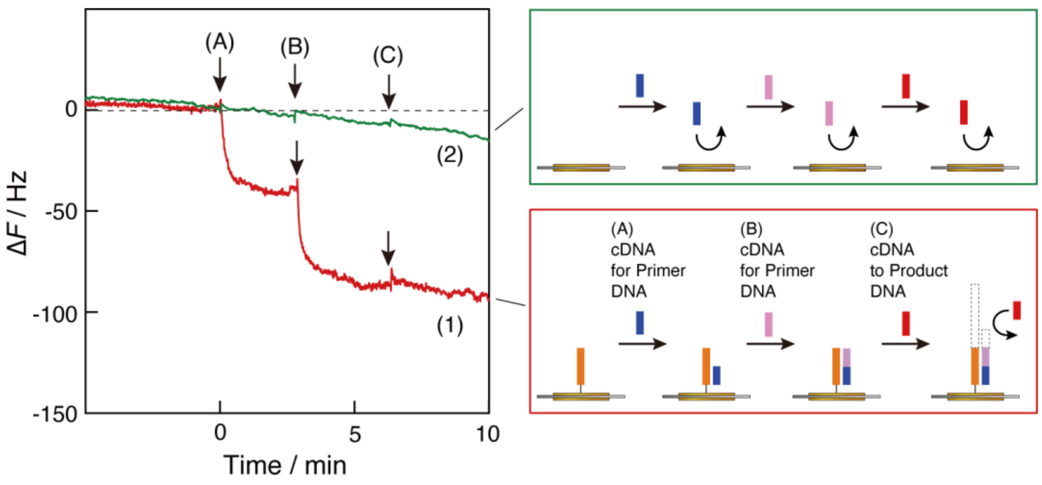

Figure S2. Confirmation of DNA elongation and non-elongation by DNA hybridization experiments on the QCM plate. The experimental condition: each [cDNA] = 50 nM at 20°C.

Experimental Information:

After the observation in Figure 6 in the text, the QCM cell was washed with 0.5 M EDTA solution. The cell was then washed with Milli-Q water and was incubated in 500 μL of 10 mM sodium hydroxide for 1 min to denature the dsDNA to ssDNA. The solution in the QCM cell was replaced with 500 μL of Hybridization buffer solution (10 mM Tris-HCl, pH 8.0, 1 mM EDTA, 0.15 M NaCl) and was set into the QCM device. After stabilization of the frequency of the QCM, the cDNAs (A), (B), and (C) were added in sequential. The used DNA sequences were as follows: (A) 5'-TTCTTCTCCATTCTG-3', (B) 5'-AATGTTTGTAATCAG-3', and (C) 5'-GTGCAATTTGCGGCC-3'.

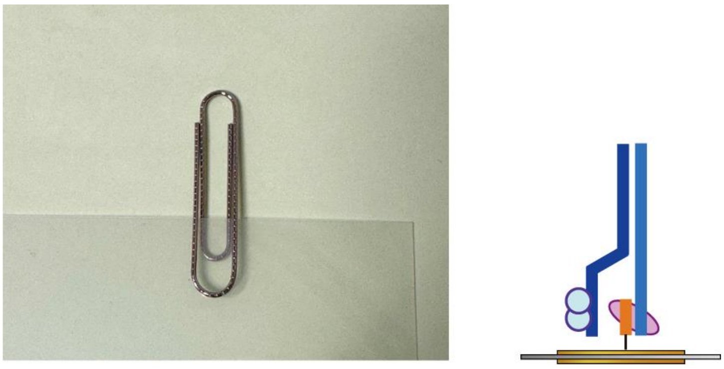

Figure S3. Image of a paperclip, which can reversibly bind to and dissociate from a paper, and illustration of the paperclip-like complex.

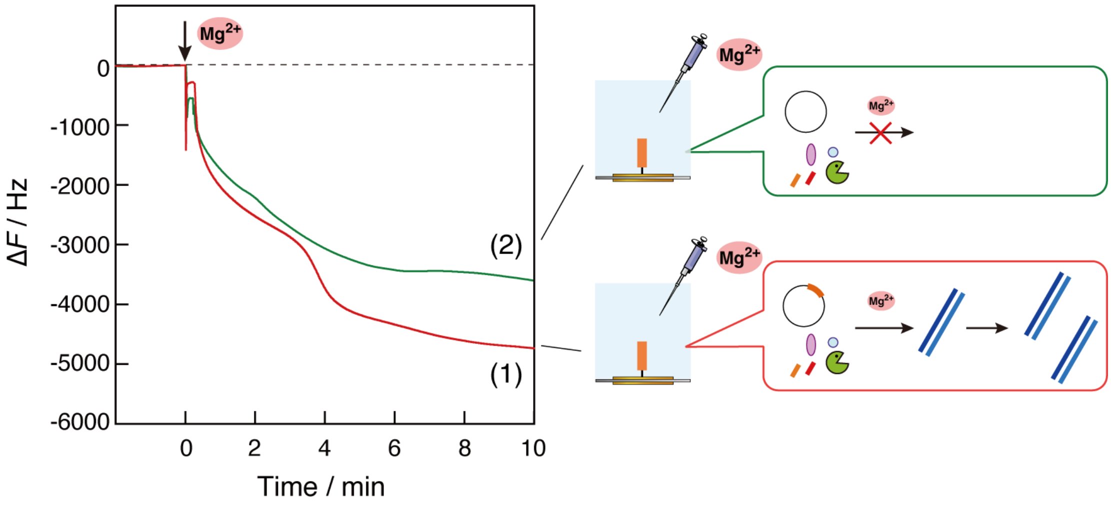

Figure S4. Time courses of the frequency changes (ΔF) of RPA reaction monitoring in response to the addition of Mg^{2+} ions into the QCM cell in the presence of (1) a template DNA matching the primers and (2) a template DNA that did not match the primers. pGEX-2T bacterial vector for expressing GST fusion proteins (GE Healthcare Technologies Inc.) was used in this experiment to investigate the specificity of RPA reactions.

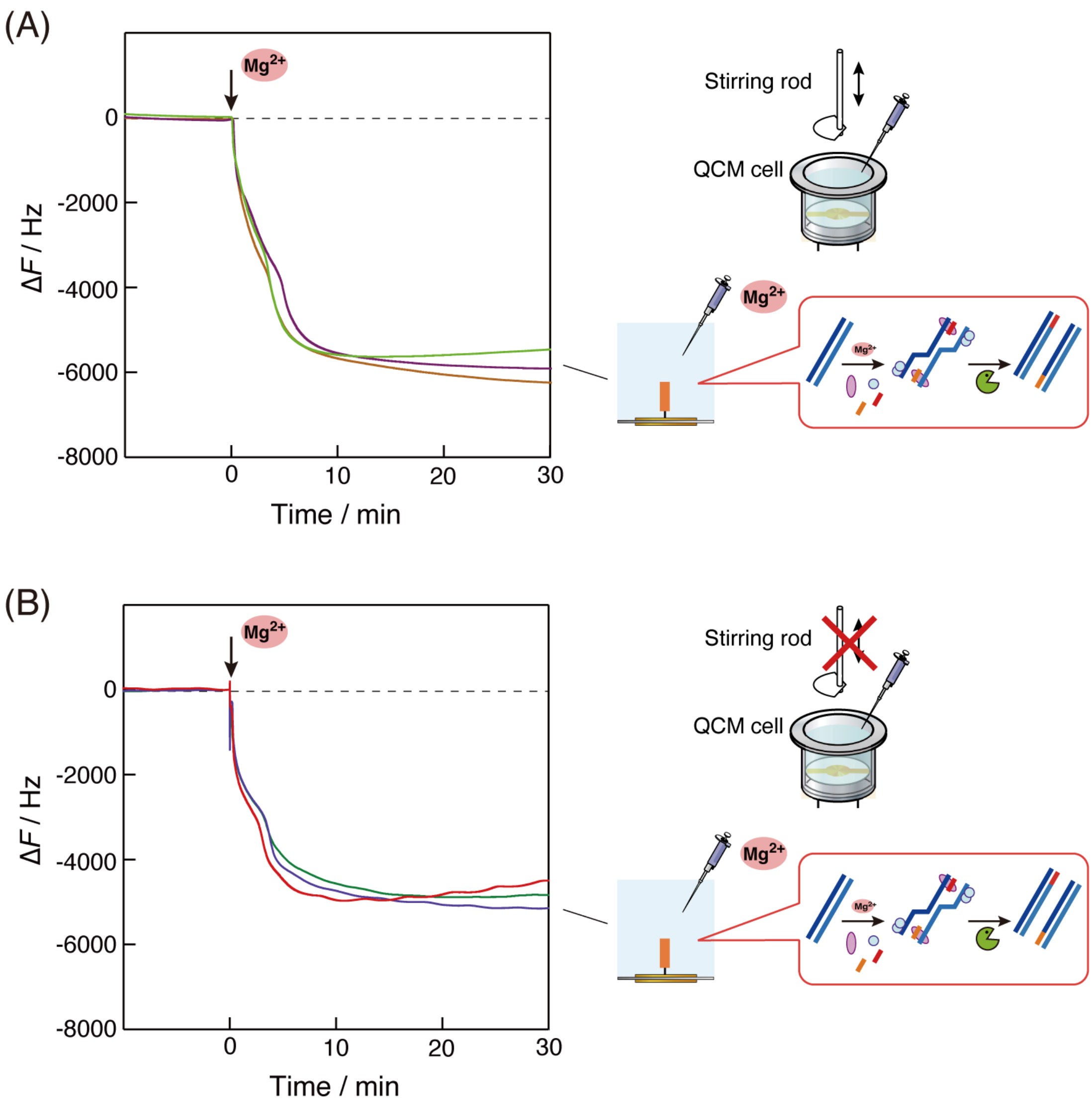

Figure S5. Repetitive validations of time courses of the frequency changes (ΔF) of RPA reaction monitoring in response to the addition of Mg^{2+} ions into the QCM cell (A) using the stirring function with the stirring rod in the same condition as curve (1) in Figure 2 and (B) without using the stirring function but instead using a pipetting operation in the QCM device in the same condition as the curve of Figure 7a.
